# Synthetic X-Q space learning for diffusion MRI parameter estimation: a pilot study in breast DKI

**DOI:** 10.1007/s11548-025-03550-7

**Published:** 2025-11-24

**Authors:** Yoshitaka Masutani, Kousei Konya, Erina Kato, Naoko Mori, Hideki Ota, Shunji Mugikura, Kei Takase, Yuki Ichinoseki

**Affiliations:** 1 Department of Medical Image Computation, Tohoku University Graduate School of Medicine, Sendai, Japan; 2 Department of Diagnostic Radiology, Tohoku University Hospital, Sendai, Japan; 3 Department of Radiology, Akita University Graduate School of Medicine, Akita, Japan; 4 Division of Image Statistics, Tohoku Medical Megabank Organization, Tohoku University, Sendai, Japan

**Keywords:** Diffusion MRI, Model parameter estimation, Machine learning, Diffusional kurtosis imaging (DKI), Breast MRI

## Abstract

**Purpose:**

For diffusion MRI (dMRI) parameter estimation, machine-learning approaches have shown promising results so far including the synthetic Q-space learning (synQSL) based on regressor training with only synthetic data. In this study, we aimed at the development of a new method named synthetic X-Q space learning (synXQSL) to improve robustness and investigated the basic characteristics.

**Methods:**

For training data, local parameter patterns of 3 × 3 voxels were synthesized by a linear combination of six bases, in which parameters are estimated at the center voxel. We prepared three types of local patterns by choosing the number of bases: flat, linear and quadratic. Then, at each location of 3 × 3 voxels, signal values of the diffusion-weighted image were computed by the signal model equation for diffusional kurtosis imaging and Rician noise simulation. The multi-layer perceptron was used for parameter estimation and was trained for each parameter with various noise levels. The level is controlled by a noise ratio defined as a fraction of the standard deviation in the Rician noise distribution normalized by the average *b* = 0 signal values. Experiments for visual and quantitative validation were performed with synthetic data, a digital phantom and clinical breast datasets in comparison with the previous methods.

**Results:**

By using synthetic datasets, synXQSL outperformed synQSL in the parameter estimation of noisy data sets. Through the digital phantom experiments, the combination of synXQSL bases yields different results and a quadratic pattern could be the reasonable choice. The clinical data experiments indicate that synXQSL suppresses noises in estimated parameter maps and consequently brings higher contrast.

**Conclusion:**

The basic characteristics of synXQSL were investigated by using various types of datasets. The results indicate that synXQSL with the appropriate choice of bases in training data synthesis has the potential to improve dMRI parameters in noisy datasets.

**Supplementary Information:**

The online version contains supplementary material available at 10.1007/s11548-025-03550-7.

## Introduction

Diffusion MRI (dMRI) quantitatively characterizes the local properties of microstructures in living organisms based on measurement of water molecule displacement [1]. A dMRI acquisition consists of diffusion-weighted imaging (DWI) with multiple directions and strengths of the gradient field, which is called motion probing gradient (MPG). Then, quantitative information is provided as dMRI signal model parameters for diffusion-related physical information and anatomical information. So far, a lot of models have been proposed for various purposes including the simplest model for Gaussian diffusion, which was initiated by Stejskal and Tanner [2], which provides the diffusion coefficient as a unique parameter. In addition, the diffusional kurtosis imaging (DKI) model quantifies the non-Gaussian diffusion due to water diffusion restricted by microstructures [3], and the diffusion tensor imaging (DTI) model [4] evaluates anisotropic 3D Gaussian diffusion by second-order tensor. Also, neurite orientation dispersion and density imaging (NODDI) [5], soma and neurite density imaging (SANDI) [6], and AxCaliber [7] are known as models providing geometric features of microanatomy.

Conventionally, the parameters have been obtained by model fitting to real DWI data by numerical methods [8] including least-squares fitting (LSF). Generally, a more complex signal model requires more computational cost. Recently, machine-learning (ML) techniques opened a new horizon, which construct regressor models that link DWI signals and model parameters directly. Golkov et al. proposed an ML-based method called q-space deep learning based on real DWI data with fitting results and reported its advantages of fast computation [9]. However, there exist two major important limitations of ML-based regressors based on real data. First, diversity with a large quantity of training data is required, which is often missed with datasets of only healthy subjects [10]. Second, the quality of the fitting results for training is critical. To overcome these problems, methods using synthetic training data were proposed [6, 10–14], which we call *synthetic q-space learning* (synQSL). In the process of training data synthesis, dMRI parameters are generated as random values, and then converted to DWI signals by model equations with simulated noise. A big advantage of the approach is that there is no limit for training data in quantity and variety, though the simulated noise must be carefully adjusted [12]. Also, this approach can refer to the gold standard synthetically generated unlike fitting and can achieve certain robustness.

Basically, dMRI parameters are estimated in a voxel-by-voxel manner to obtain local features. For the spatial smoothness of estimated parameter maps, however, certain regularization mechanisms are often introduced in fitting approaches for higher robustness. It is natural to consider such a regularization factor in the synthetic ML-based approach for dMRI parameter estimation. One of the reasonable approaches to introduce regularization into synQSL is the additional use of adjacent voxel values of DWI for regressor input, for which we named *synthetic X-Q space learning* (synXQSL). That is, machine learning is performed in X-Q space as a joint space of voxel location and diffusion weighting. For the approach, we need to synthesize training data including signal values of adjacent voxels with certain regularity, that is, spatial smoothness as observed in real data. The simplest assumption is uniformity; that means the parameter value is uniform in the adjacent region, which is expected to yield too smooth estimation results with edge blurring. Therefore, a challenge of training data generation for synXQSL is to synthesize reasonable sets of local patterns of dMRI parameters for higher robustness by spatial regularization.

Based on the above, the purpose of this study is focused on the development of a new ML-based dMRI parameter estimation scheme of synXQSL. As a dMRI signal model, we used DKI and clinical breast dMRI datasets are used for the validation in addition to the digital phantom.

## Methods

First, formulation for Q-space and X-Q space are shortly given here. The 3D location at Q-space is defined by Q-vector: ${\mathbf{q}}$ representing strength and direction of diffusion weighting by MPG for a single dMRI data acquisition [1]. On the other hand, X-space is used for representing the 3D location of voxels ${\mathbf{x}}$ in the physical space. Therefore, signal values of dMRI data in X-Q space can be expressed as $S\left( {{\mathbf{x}},{\mathbf{q}}} \right)$. That is, X-Q space is defined as a joint space representing voxel location and diffusion weighting strength and direction. In this study, the range of X-space is limited to 2D plane due to 2D DWI acquisition and to the extent of 3 × 3 voxels. Q-space is also limited to 1D due to the assumption of isotropic diffusion in the breast dMRI acquisition in this study. Therefore, we use $S\left( {{\mathbf{x}},b} \right)$ with scaler b-values for MPG strength for X-Q space signal. The DKI signal model equation by Jensen et al. [3] is formulated as below.

1 $$ S\left( {{\mathbf{x}},b} \right) = F\left( {b,{\mathbf{p}}\left( {\mathbf{x}} \right)} \right) = S_{0} \left( {\mathbf{x}} \right){\text{ exp}}\left( { - bD\left( {\mathbf{x}} \right) + \frac{{b^{2} D\left( {\mathbf{x}} \right)^{2} K\left( {\mathbf{x}} \right)}}{6}} \right) $$

**Fig. 1:**
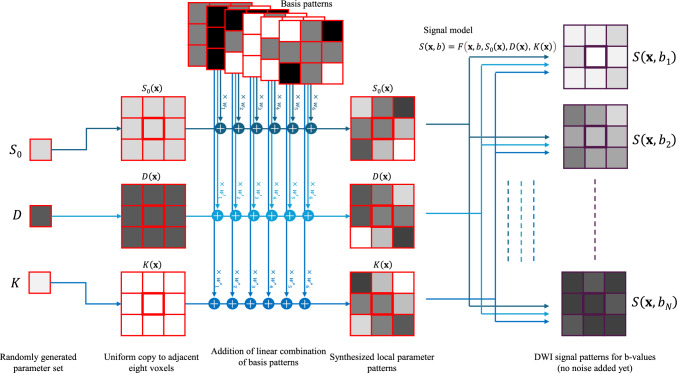
Schematic diagram of pipeline for training data synthesis in synXQSL before noise contamination. Note that linear combination weights $w$ are generated independently for each parameter and each basis

$F\left( \cdot \right)$ is the signal model function of which value is determined by b-value and model parameters ${\mathbf{p}}$ at voxel location ${\mathbf{x}}$. The parameter set ${\mathbf{p}}\left( {\mathbf{x}} \right) = \left( {S_{0} \left( {\mathbf{x}} \right),D\left( {\mathbf{x}} \right),K\left( {\mathbf{x}} \right)} \right)$ includes $S_{0}$ as the ideal baseline signal value at $b = 0$, $D$ as the diffusion coefficient for the magnitude of diffusion, and $K$ as the diffusional kurtosis for non-Gaussianity quantification. Training data synthesis is performed in the following steps (see also Fig. 1).

(1) Random parameter generation

Parameter set of $S_{0}$, $D$ and $K$ are generated as uniform random numbers in the condition shown in Table 1 by using the Mersenne twister (MT) algorithm [15]. The constraint of s $D\, \cdot \,K < 3/b_{\max }$ guarantees the generated parameter sets satisfy monotonous signal decrease until the maximum b-value; $b_{\max }$ [3, 16]. The two values of $D$ and $K$ generated in this step are used as the target of parameter estimation, i.e., the gold standard.

**Table 1:** Ranges of parameters for DKI model and synXQSL randomly generated in uniform distribution

Parameter	Min	Max	Notes
$S_{0}$	500	8000	
$D$	0.2	6.0	(scaled)$\times 10^{ - 3} \,{\text{mm}}^{2} /{\text{s}}$
$K$	0.0	4.0	
$D\, \cdot \,K$	0.0	$3.0/b_{\max }$	(scaled)$\times 10^{ - 3} \,{\text{mm}}^{2} /{\text{s}}$
$w_{i}$ for ${\varvec{S}}_{0}$	− 4000	4000	$i = 1 \ldots M$
$w_{i} {\text{ for }}{\mathbf{D}}$	− 0.5	0.5	$i = 1 \ldots M$
$w_{i} {\text{ for }}{\mathbf{K}}$	− 0.5	0.5	$i = 1 \ldots M$

(2) Converting single parameter value to local pattern

**Fig. 2:**
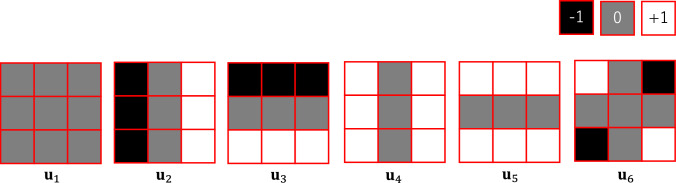
Local 3 × 3 pattern bases for parameter map synthesis corresponding to basis vectors ${\mathbf{u}}_{1} \sim {\mathbf{u}}_{6}$ of Eq. 2

In our preliminary study [17, 18], principal component analysis of real dMRI parameter map showed that small number of components can reconstruct the original pattern with correlation among parameters (supplemental material #1). For the diversity of training data, however, we determined to synthesize patterns for each parameter independently in this study. We used linear combinations of 3 × 3 basis patterns as shown in Fig. 2. The bases, ${\mathbf{u}}_{i} \user2{ }\left( {i = 1 \ldots 6} \right)$ are expressed as vectors of concatenated rows from 3 × 3 matrices as follows.

2 $$ \begin{aligned} & {\mathbf{u}}_{1} = \left( {\begin{array}{*{20}c} {\begin{array}{*{20}c} { + 0} & { + 0} & { + 0} \\ \end{array} } & {\begin{array}{*{20}c} { + 0} & { + 0} & { + 0} \\ \end{array} } & {\begin{array}{*{20}c} { + 0} & { + 0} & { + 0} \\ \end{array} } \\ \end{array} } \right),\user2{ } \\ & {\mathbf{u}}_{2} = \left( {\begin{array}{*{20}c} {\begin{array}{*{20}c} { - 1} & { + 0} & { + 1} \\ \end{array} } & {\begin{array}{*{20}c} { - 1} & { + 0} & { + 1} \\ \end{array} } & {\begin{array}{*{20}c} { - 1} & { + 0} & { + 1} \\ \end{array} } \\ \end{array} } \right),\user2{ } \\ & {\mathbf{u}}_{3} = \left( {\begin{array}{*{20}c} {\begin{array}{*{20}c} { - 1} & { - 1} & { - 1} \\ \end{array} } & {\begin{array}{*{20}c} { + 0} & { + 0} & { + 0} \\ \end{array} } & {\begin{array}{*{20}c} { + 1} & { + 1} & { + 1} \\ \end{array} } \\ \end{array} } \right),\user2{ } \\ & {\mathbf{u}}_{4} = \left( { + \begin{array}{*{20}c} {\begin{array}{*{20}c} 1 & { + 0} & { + 1} \\ \end{array} } & {\begin{array}{*{20}c} { + 1} & { + 0} & { + 1} \\ \end{array} } & {\begin{array}{*{20}c} { + 1} & { + 0} & { + 1} \\ \end{array} } \\ \end{array} } \right),\user2{ } \\ & {\mathbf{u}}_{5} = \left( {\begin{array}{*{20}c} {\begin{array}{*{20}c} { + 1} & { + 1} & { + 1} \\ \end{array} } & {\begin{array}{*{20}c} { + 0} & { + 0} & { + 0} \\ \end{array} } & {\begin{array}{*{20}c} { + 1} & { + 1} & { + 1} \\ \end{array} } \\ \end{array} } \right)\;{\text{and}} \\ & {\mathbf{u}}_{6} = \left( {\begin{array}{*{20}c} {\begin{array}{*{20}c} { + 1} & { + 0} & { - 1} \\ \end{array} } & {\begin{array}{*{20}c} { + 0} & { + 0} & { + 0} \\ \end{array} } & {\begin{array}{*{20}c} { - 1} & { + 0} & { + 1} \\ \end{array} } \\ \end{array} } \right). \\ \end{aligned} $$

In this study, we examined three types of pattern synthesis by the combination of the bases. The first pattern consisting of only ${\mathbf{u}}_{1}$ assumes uniform signals. The next is by linear combination of ${\mathbf{u}}_{1} \sim {\mathbf{u}}_{3}$ and assumes spatial signal transition is linear. Lastly, the quadratic pattern consists of ${\mathbf{u}}_{1} \sim {\mathbf{u}}_{6}$. The weights for the linear combinations are also generated by the MT algorithm as uniform random values with the check of monotonous signal decrease at all the nine locations.

(3) Voxel-by-voxel DWI synthesis with adding noise

Once $S_{0}$, $D$ and $K$ parameters are determined at each location within 3 × 3 pattern, series of DWI signals for b-values are computed based on the signal model in Eq. 1. Then, random noise is added at each X-Q space location. We used the simple Rician noise model [3] as,

3 $$ S^{\prime} = F_{\eta } \left( {b,{\mathbf{p}},\sigma } \right) = \sqrt {S^{2} + N\left( {0,\sigma } \right)^{2} } {,} $$

where $S^{\prime}$ is the noise-corrupted value, $S$ is the original value of ( Eq. 1) , and $N\left( {0,\sigma } \right)$ is the zero-mean Gaussian random value with a standard deviation of $\sigma .$ Amount of noise for training or test is controlled by the noise ratio, $NR = \sigma /\overline{{S_{0} }}$, in which $\overline{{S_{0} }}$ indicates the average value of the baseline signal $S_{0}$.

For the summary of the data synthesis, a set of X-Q space data is formulated below. For 3 × 3 pattern of diffusion coefficient ${\mathbf{D}}$ as a vector of nine elements,

4 $$ {\mathbf{D}} = \left( {\begin{array}{*{20}c} {D_{0} } & \cdots & {D_{8} } \\ \end{array} } \right) = {\mathbf{D}}_{0} + \mathop \sum \limits_{i = 1}^{M} w_{i} {\mathbf{u}}_{i} {,} $$

where $D_{i}$ is the diffusion coefficient at the $i$-th location (note that center is indexed as 0), ${\mathbf{D}}_{0}$ denotes a vector with identical nine elements of $D_{0}$, $M$ is the number of bases, and $w_{i}$ is weight for linear combination. The range of $w_{i}$ was experimentally determined for each parameter shown in Table 1. In this study, $M = 6$ (quadratic) is default and is compared with $M = 1$ (flat) and $M = 3$ (linear) in the experiments. Patterns of other parameters, ${\mathbf{S}}_{0}$ and ${\mathbf{K}}$ are also generated in the same way. For the series of signals at nine locations and for $N$ b-values; $S_{i, j}$ is defined as,

5 $$ S_{i, j} = F_{\eta } \left( {b_{j} ,{\mathbf{p}}\left( {{\mathbf{x}}_{i} } \right),\sigma } \right)\quad \left\{ {i = 0 \ldots 8, j = 0 \ldots N} \right\}{.} $$

The parameter set $${\mathbf{p}}\left( {{\mathbf{x}}_{i} } \right)$$ includes $$S_{0}$$, $$D$$ and $$K$$ at the $$i$$-th location $${\mathbf{x}}_{i}$$. In this study, we assume a single $$ b = 0$$ acquisition ($$b_{0} = 0$$), and therefore the number of non-zero b-values is $$N$$ ($$b_{j} \ne 0;j > 0$$). Finally, 10^6^ sets of X-Q space data are prepared each for training, validation and testing in this study.

For the regressor, multi-layer perceptron (MLP) to output single parameter was used in this study for comparison with the previous synQSL [12]. To clarify the effect of additional input of adjacent voxel values, we used the same architecture as synQSL. That is, the trained MLP outputs an estimated value of single parameter ($D$ or $K$) against the $9N$ inputs of signal decay ratios $E_{i, j} = S_{i, j} /S_{i, 0}$ $\left\{ {i = 0 \ldots 8, j = 1 \ldots N} \right\}$, which are the signal values for $b \ne 0 \left\{ {j = 1 \ldots N} \right\}$ normalized by those for $b = 0 \left\{ {j = 0} \right\}$ at the same voxel location. Through the training, hyperparameters such as number of mid-layers, number of units, and so on are adjusted with validation dataset. Specifically, the optimal set of hyperparameters yielding the minimum RMSE in estimating parameters for the validation datasets is searched by a brute force method. The search range is given with the following combinations: 3, 4, or 5 for the number of mid-layers; 64, 128, or 256 for the number of units per mid-layer; 0.0, 0.01, or 0.1 for the dropout ratio; 50, 100, or 150 for the number of training epochs; and 1,000, 10,000, or 100,000 for the batch number (supplementary materials #2–7).

**Fig. 3:**
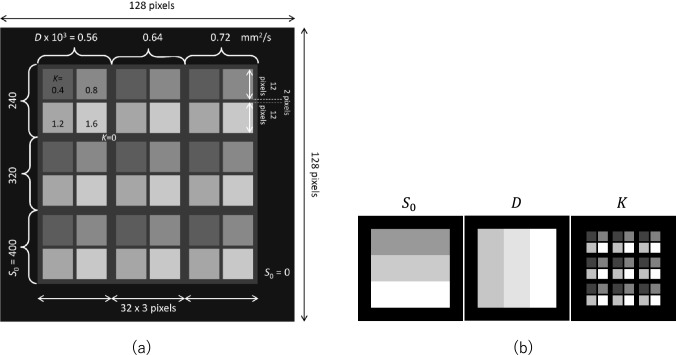
Digital phantom of 128 × 128 × 128 (an axial slice is shown). **a** Parameter layout, and **b** each parameter

We performed the experiments of DKI parameter estimation for comparing three estimation methods, LSF, synQSL and synXQSL. We used three types of dataset, the synthetic test data sets generated in the above procedure, a digital phantom [19], and clinical datasets of breast dMRI. The combination of b-values for the synthetic test data and the digital phantom was identical to that of the clinical datasets. First, we prepared synthetic data of noise ratios $NR = 0.0,{ }0.001,{ }0.01,{\text{ and }}0.1$ for cross-tests among the noise levels, in which all the combination of noise levels for training and testing were examined. For the clinical dMRI data, $NR = 0.1$ were estimated as the matched noise level by a preliminary experiment, in which standard deviation at the uniform area in $b = 0$ image was estimated. The digital phantom has the dimension of 128 × 128 × 128, in which uniform regions of three variations of $S_{0}$, three variations of $D$ and five variations of $K$ are assigned (Fig. 3). By using Rician noise of $\sigma = 32.0$, the noise level of the phantom was adjusted to obtain $NR = 0.1$ at the area of $S_{0} = 320$. The clinical dMRI datasets of 30 cases (26 malignant and 4 benign) were consecutively obtained by 2D echo planar imaging (EPI) sequence in a 3T clinical MRI scanner (Philips Achiva dStream) with the clinical routine settings (Table 2 and supplementary material #7). The datasets were retrospectively collected in Tohoku University Hospital, and the use of the datasets for this study was approved by the internal ethics review board (registration #: 2023-1-804).

**Table 2:** Clinical dMRI acquisition settings and parameters

Sequence	2D EPI-SE (Fat Sat IR)
TR/TE $\left( {{\text{ms}}} \right)$	5300/80
FOV ${ }\left( {{\text{mm}}^{2} } \right)$	180 × 180
Matrix size	256 × 256
Number of slices	25
Slice thickness ${ }\left( {{\text{mm}}} \right)$	5
*b*-values $\left( {{\text{s}}/{\text{mm}}^{2} } \right)$	0, 50, 850, 1000, 1500, 2000, and 2500
Number of excitations	2

For quantitative comparison between synQSL and synXQSL, several metrics such as RMS error (RMSE), SNR (signal-to-noise ratio), CNR (contrast-to-noise ratio) and SSIM (structural similarity index measure) were measured in the digital phantom and the clinical images. For the digital phantom with the ground truth, the indices of RMSE and SNR were obtained at the regions of $K \ne 0$. The CNR values were obtained between the foreground at the $K \ne 0$ regions and the background regions of $K = 0$ and $D \ne 0$. The SSIM values were calculated between the maps of the ground truth and the estimated parameter maps in the entire image area except for the region of $S_{0} = 0$. For the clinical datasets without ground truth, only the CNR values were measured between the foreground at the mammary tissues and the background at the fat tissues because the breast dMRI has too narrow uniform area to evaluate SNR except around the background fat tissues. All the index values were calculated with the standard function in Image J (National Institutes of Health and the Image J community). The details of masks used for measuring the metrics are shown in the figures of the supplementary material #8. For the statistical comparison, the Wilcoxon signed-rank tests ($p = 0.05$ for significance) were performed for the index values.

## Results

**Fig. 4:**
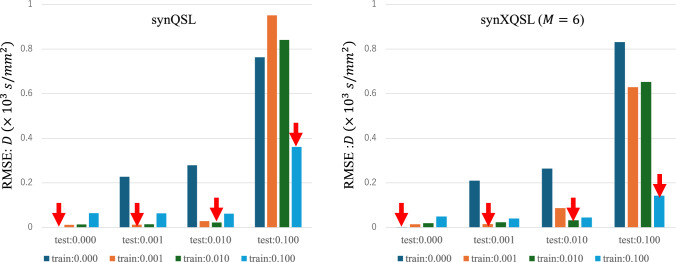
Estimation RMSE: $D \,\left( { \times 10^{3} \,{\text{s/mm}}^{2} } \right)$. Cross-tests among four levels of noise for training and test. The label “test:xxx” and “train:yyy” mean the estimation result of the test datasets with noise ratio of xxx by the regressor trained by datasets with noise ratio of yyy

**Fig. 5:**
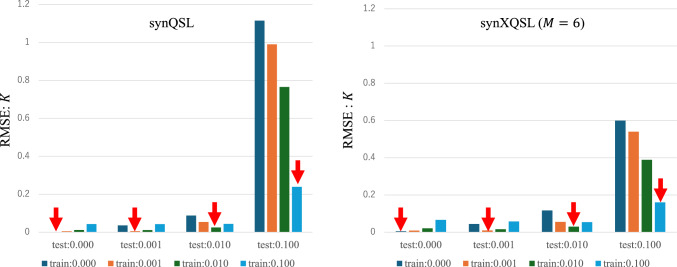
Estimation RMSE: $K$. Cross-tests among four levels of noise for training and test. The label “test:xxx” and “train:yyy” are same as those of Fig. 4

Figures 4 and 5 show the parameter estimation results by synQSL and synXQSL, $D$ (10^3^-fold) and $K$ for synthetic data of various noise ratios, in which root-mean square error (RMSE) is displayed for each noise ratio combination between training (including validation) and test. Note that only Q-space data at the center location is used in synQSL. For synXQSL, all the bases are used for training of quadratic patterns. The synXQSL outperformed synQSL and LSF (supplementary material #9 and 10), especially for higher noise ratio of 0.1. As reported in the previous study [12], noise level matching is important also in synXQSL (red arrows). The $K$ parameter is improved more than $D$.

**Fig. 6:**
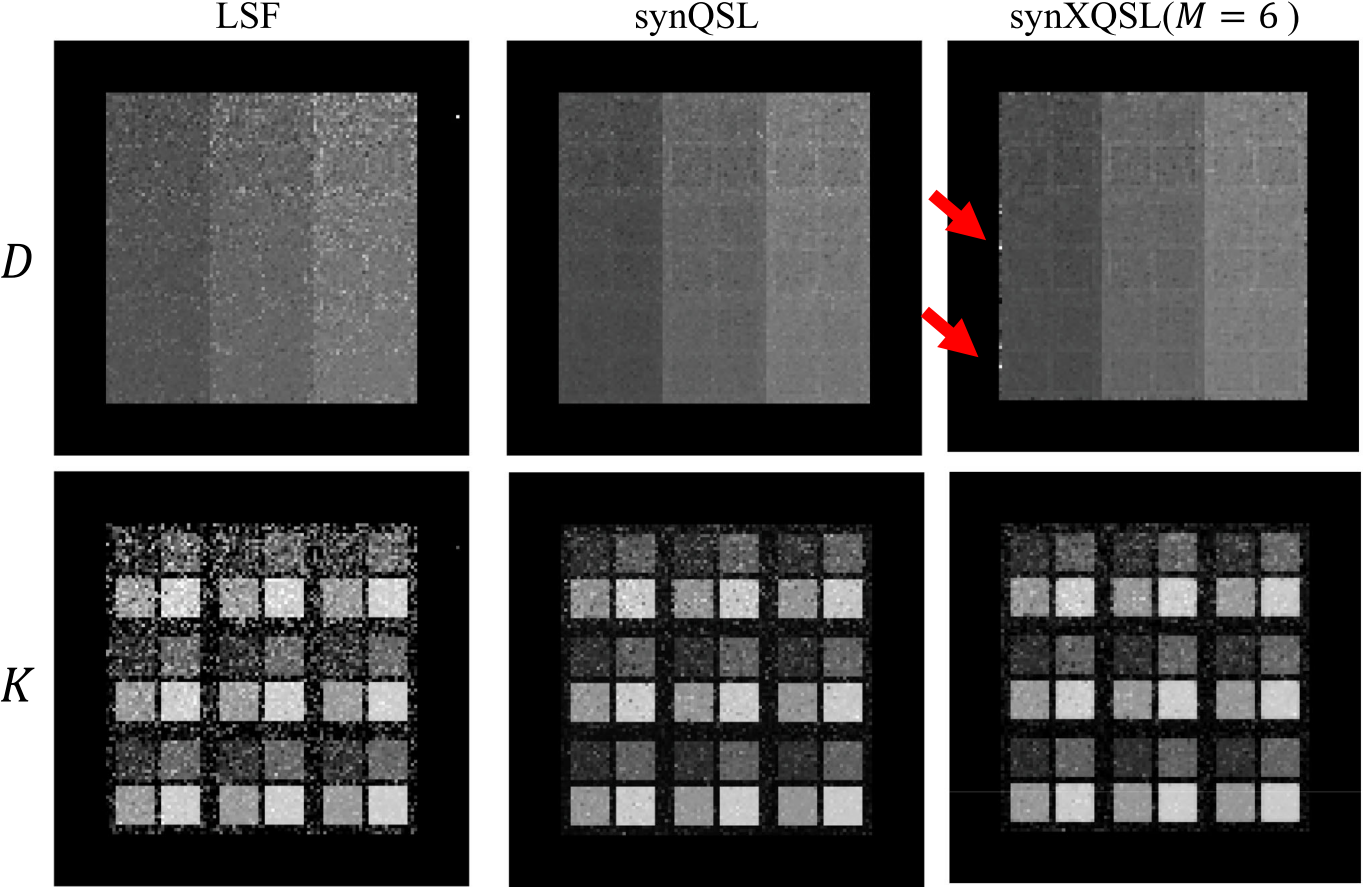
Parameter maps of digital phantom estimated by LSF, synQSL and synXQSL. $D$ (upper) and $K$ (lower)

**Fig. 7:**
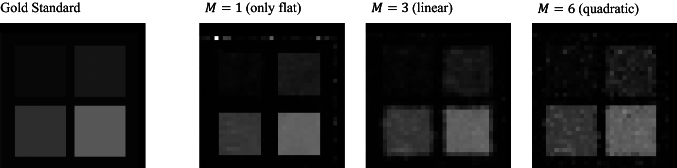
Zoomed part of $K$ map estimated by synXQSL with three basis combination types

**Fig. 8:**
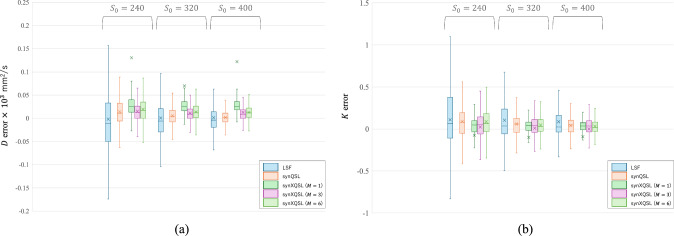
Boxplots of parameter estimation error in digital phantom by LSF, synQSL and synXQSL. $D$ (left) and $K$ (right)

Figure 6 shows the parameter maps estimated by LSF, synQSL and synXQSL for the digital phantom. At the edge of $D$ map by synXQSL, a few voxels of large error are found in the slice (red arrow), which are located at the border between zero and non-zero values of $S_{0}$. The result also indicates that more errors are observed in the low $S_{0}$ areas where noise level became relatively high. In Fig. 7, a comparison among the number of bases used in synXQSL is shown with the zoomed $K$ map around the upper right corner of the phantom. The result indicates the error at the border is critical when less bases are used, that is, the square shapes are eroded with only the flat basis and edge blurring is observed in the linear bases result. However, smoothness inside the uniform square pattern is better with less bases. For quantitative evaluation, Fig. 8 shows boxplots (without outliers) of signed error for $D$ and $K$ estimation by LSF, synQSL and synXQSL with three types of basis combination. The further details of errors are also summarized in Table 3 with the errors of root-mean square (RMS), minimum and maximum. Due to the large errors (outliers) at the pattern boundaries, the average errors shown by cross-marks are shifted to outside of the boxes in the synXQSL ($M = 1$) results. Though the amounts of $D$ errors are similar among the three methods, $K$ results clearly show the advantage of ML-based methods, especially for synXQSL ($M = 6$), which yielded less RMS errors and smaller error ranges. For the $D$ maps, only CNR showed significant difference ($p = 3.4 \times 10^{ - 6}$) to indicate the improvement by synXQSL ($M = 6$) while the other indices for the $K$ maps showed clear improvement by synXQSL ($M = 6$) with significant difference ($p = 0.00015$ for RMSE, $p = 0.0006$ for SNR and $p = 0.001$ for CNR). The SSIM indices against the ground truth showed similar values in $D$ maps; 0.997 for both methods while the values for $K$ maps; 0.599 for synXQSL ($M = 6$) and 0.589 in synQSL imply slight improvement by synXQSL.

**Table 3:** Quantitative comparison of parameter estimation in digital phantom among LSF, synQSL and synXQSL with three basis combination types

	$S_{0} = 240$						$S_{0} = 320$						$S_{0} = 400$					
	$D \times 10^{3}$ mm^2^/s			$K$			$D \times 10^{3}$ mm^2^/s			$K$			$D \times 10^{3}$ mm^2^/s			$K$		
	RMS	min	max	RMS	min	max	RMS	min	max	RMS	min	max	RMS	min	max	RMS	min	max
LSF	0.084	− 0.50	0.65	0.57	− 50.66	2.10	0.056	− 0.32	0.48	0.35	− 5.72	1.73	0.039	− 0.26	0.38	0.26	− 2.63	1.55
synQSL	0.038	− 0.21	**0.47**	0.23	− 1.37	1.80	**0.024**	**− 0.14**	**0.21**	0.16	− 1.27	1.44	0.020	**− 0.12**	**0.21**	0.12	− 0.68	**1.12**
*synXQSL*																		
$M = 1$	0.600	− 0.30	10.0	0.46	− 1.70	15.63	0.380	− 0.42	10.0	0.43	− 1.66	9.32	0.560	− 0.62	10.0	0.45	− 2.86	9.38
$M = 3$	**0.043**	**− 0.10**	4.0	0.21	− 1.25	**1.55**	0.030	− 0.85	4.0	0.19	− 1.24	**0.96**	0.044	− 0.65	5.0	0.18	− 1.22	1.31
$M = 6$	0.044	− 0.82	4.0	**0.20**	**− 1.014**	1.63	0.029	− 0.60	6.0	**0.14**	**− 0.88**	1.31	**0.030**	− 0.57	4.0	**0.11**	**− 0.73**	1.56

For each $S_{0}$ level and parameter, the most desirable is shown in bold.

**Fig. 9:**
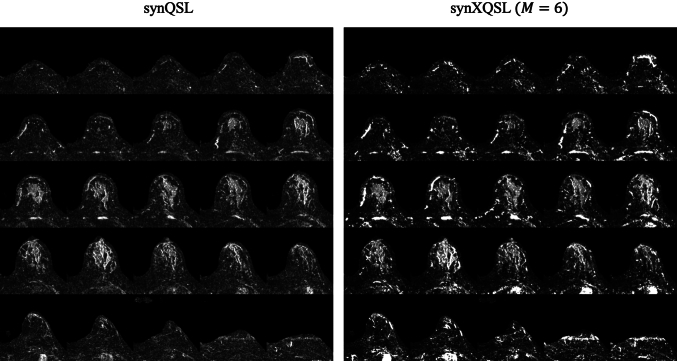
Parameter maps of clinical data estimated: diffusion coefficient $D$: synQSL (left) and synXQSL (right)

**Fig. 10:**
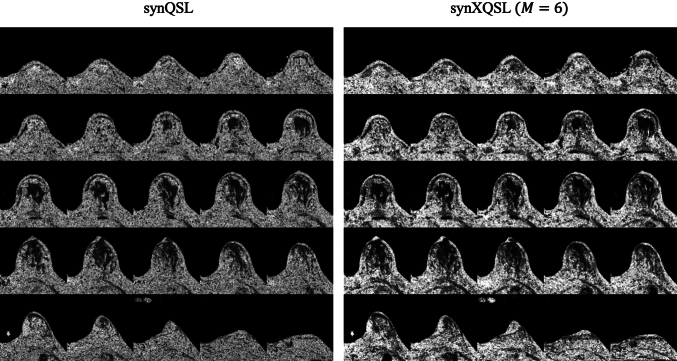
Parameter maps of clinical data estimated: diffusional kurtosis $K$: synQSL (left) and synXQSL (right)

**Fig. 11:**
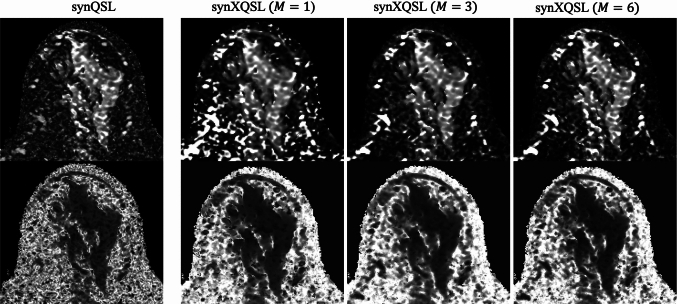
Zoomed parameter maps of clinical data: $D$ (upper) and $K$ (lower). (from left) synQSL, synXQSL ($M = 1$), synXQSL ($M = 3$), and synXQSL ($M = 6$)

Figures 9 and 10 show the entire maps for $D$ and $K$ by synQSL and synXQSL for the clinical datasets. As a whole, similar results but higher contrast in synXQSL are observed. Details including three types of synXQSL bases are displayed in Fig. 11 with the zoomed maps of a slice. The result clearly shows the difference in depicting the structures of the mammary gland, the adipose tissues and the vasculature. First, in the $D$ map by synQSL, white dots are found which are thought to be by an overestimation error due to voxel-by-voxel manner of estimation, while synXQSL yielded no such errors in the $D$ maps. Second, vasculatures are depicted in higher values in the $D$ maps by synXQSL than those by synQSL, where perfusion component equivalent to high diffusion coefficient is generally dominative. Such tendency is more noticeable in less bases of synXQSL. Next, in the $K$ maps, smoother results were obtained by synXQSL, while the result by synQSL seems to include more errors of negative $K$ values as black dots at the adipose tissue. Also in the mammary gland, smoother appearance was observed in synXQSL while keeping the fine structures of low $K$ values. Regarding the $K$ maps, the difference by the number of synXQSL bases was not significant. The CNR values between the mammary tissue and the surrounding fat tissues were 1.685 for synXQSL ($M = 6$) and 1.533 for synQSL in the $D$ maps, and 3.714 for synXQSL ($M = 6$) and 2.233 for synQSL in the $K$ maps. These values imply that synXQSL can improve the contrast in parameter maps by real data.

In terms of computation time, synXQSL required 2.5–3.0 times longer than LSF and synQSL. Please note that the computation of LSF is optimized with the SIMD (single instruction, multiple data) and multi-thread framework in a PC workstation with a standard CPU (Intel Core i5) while synQSL and synXQSL computation were performed with GPU (NVIDIA A100).

## Discussion

Through the experiments, basic characteristics of synXQSL were revealed. The importance of noise level matching [12] was confirmed also for synXQSL. A certain advantage against synQSL was observed when we estimate parameters in noisy data such as clinical dMRI used in this study. However, as shown in the synthetic data experiments (Figs. 4, 5), synXQSL might not be advantageous for less noisy data ($NR < 0.1$). This situation is invoked also with denoise preprocessing [20] or from AI-based reconstruction [21], and it implies that synQSL is still a choice for the cases. Especially, it was reported that AI-based reconstruction showed improvements in DWI but not in parameter map of apparent diffusion coefficient [22]. Therefore, it is expected that extensive studies reveal the true value of AI‑reconstructed DWI in dMRI model parameter estimation. Another factor is sampling density in Q-space, i.e., number of b-values. synXQSL would be effective for datasets of less b-values. In addition, the digital phantom experiment showed that the quadratic pattern was superior to the other two, which could yield artifacts. But the linear bases can also be an alternative for the clinical dataset which hardly includes sharp edges in the phantom.

Next, we discuss competitive and/or similar approaches with the focus on the methodologies and principles. Please note that the “state-of-the-art” or “de facto standard” methods for the purpose of dMRI parameter estimation related to this study are still based on the conventional fitting which is used in cohort studies extensively. That is, ML-based dMRI methods have been investigated extensively so far and their advantages have been shown to some extent but they are not confirmed totally so that they supersede the conventional approaches. However, several related approaches discussed below can be candidates in addition to synQSL and synXQSL. The related approaches can be roughly divided into two categories: global and local approaches below. The global approach yields whole parameter maps from the entire DWI dataset input, mainly based on Convolutional Neural Networks (CNN) [23–25]. This approach, not in the sequential voxel-by-voxel estimation manner, needs datasets of whole size real DWI and consequently suffers from the problem of quantity, diversity and quality for training data, while the recent studies on generative AI [26] might make a breakthrough in the future. Methodologically, the global approach could be regarded as an extreme extension of X-space reference in addition to Q-space. This viewpoint leads to a discussion on the essential question “how large range of X-space reference should be used to improve the robustness of parameter estimation”. Originally, dMRI signal models and parameter maps are aimed at local feature quantification and therefore parameter estimation should not be influenced by information from other locations. In that sense, limiting the range of CNN to a smaller patch [25] is a better solution closer to synXQSL. In this sense, synXQSL is thought to have a certain reasonableness against the global approach based on CNN. As far as we searched the literature, a few reports were found for the local approach. Kaandorp [27] proposed a method for IVIM (Intra-Voxel Incoherent Motion) parameter estimation, in which training datasets are synthesized with all plausible combinations of neighboring correlations while synXQSL synthesizes patterns by linear combination of a limited number of bases. However, the training datasets in both methods would likely become similar if the neighbor patterns of Kaandorp’s method and the bases of synXQSL are increased. Another local approach was proposed from the same group, which utilizes attention of neighbors extended to 7 × 7 size and fractal-noise maps [28] and was reported to outperform their previous method [27]. Another transformer-based approach was proposed by Zheng, combined with dictionary-based sparse representation [29]. In Zheng’s experiments with changing patch sizes, including 3 × 3 to the whole image, the loss was minimal with 3 × 3 size. Though parameter estimation architectures are quite different from synXQSL, those results imply a cue for determining the best size for X-space reference, and further studies are expected.

This study has some limitations, which are motivations for further investigation. First, we examined only a 1D signal model of DKI in the breast region. Currently, we are investigating the IVIM-DKI model shown to be useful in the breast tumor analysis [30, 31]. Especially, breast cancer classification including subtypes is in our scope and is expected to benefit from a better parameter estimation scheme by synXQSL because more reliable parameter estimation can yield better classification by more separable distributions in feature space. In addition, applications in the brain with anisotropic structures are in our scope, which require a 3D extension of synXQSL. That is, X-space fluctuation of vector and/or tensor parameters must be expressed for data synthesis. For that purpose, Riemannian metrics [32, 33] are planned to be introduced in synXQSL. Next, we have tried only MLP as a regressor for its versatility and flexibility. We are investigating random forest [6, 34] and Kolmogorov-Arnold Network (KAN) [35, 36] for comparison with MLP. Also, against the simple noise model we used, the real noise distribution is more complex especially based on parallel imaging [37]. Currently, we are investigating a Rician model with a Gaussian noise mixture [38], with which our preliminary experiments showed promising results. Finally, as a perspective of our synthetic approach, application in the combined signal models of diffusion and relaxation is considered [39, 40], which quantify relaxation parameters such as T1 and T2 in addition to diffusion-related parameters.

## Conclusion

In this pilot study, we investigated the basic characteristics of synXQSL aimed at robust dMRI parameter estimation and showed results in the DKI model by using synthetic datasets, a digital phantom and clinical datasets. The results indicate that synXQSL is effective for noisy datasets. For training data synthesis in synXQSL, the quadratic pattern is the first choice, and the linear one could be the second for datasets without sharp edges. As future work, we are going to perform further investigation with various models, regions, and applications.

## Supplementary Information

Below is the link to the electronic supplementary material.

Supplementary file1 (PDF 785 KB)
