# Supplementary material for: Synthetic X-Q space learning for diffusion MRI parameter estimation: a pilot study in breast DKI
Source: Int J Comput Assist Radiol Surg. 2025 Nov 24;20(12):2423–35. doi: 10.1007/s11548-025-03550-7 (PMC12689713; doi:10.1007/s11548-025-03550-7)
Supplement: Supplementary file 1 — Supplementary file1 (PDF 785 KB) [file 11548_2025_3550_MOESM1_ESM.pdf]

Supplementary material for the manuscript

- Author: Masutani Y, et al.

- Title: Synthetic X-Q space Learning for diffusion MRI parameter estimation: a pilot study in breast DKI

- Software:
  - Image J for image data including pattern bases
  - Microsoft Excel for graph plots

- Contents:
  - Local Pattern Analysis of Real Data
  - Clinical dMRI Data Example
  - Training and tuning details for synQSL and synXQSL
  - Parameter estimation RMS errors by LSF for synthetic test data

#1 Local Pattern Analysis of Real Data

Example of local joint pattern analysis by PCA for NODDI parameter maps of real data [17]

- f_{iso}: volume fraction for isotropic diffusion, f_{iso}: volume fraction for intra-cellular compartment, OD: orientation dispersion.

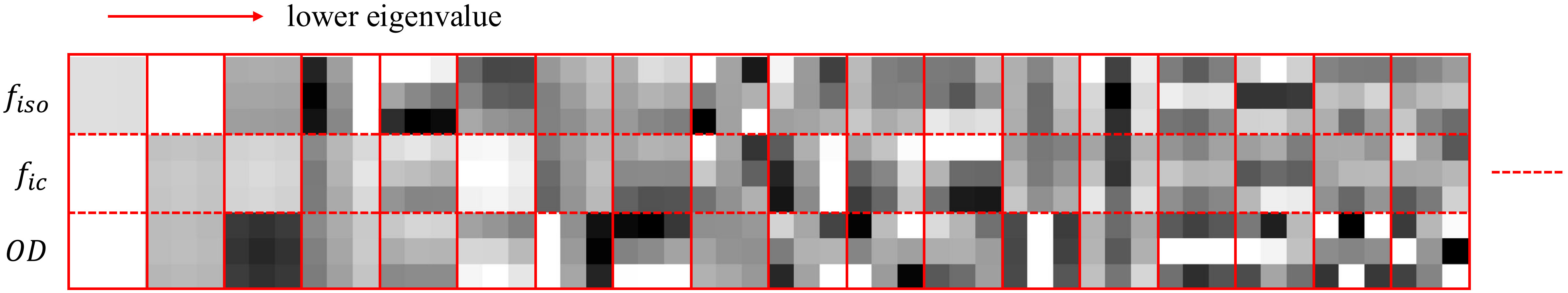

#2 Training and Tuning: Settings

Model & Data

- Model
  - DKI 1D
- MPG
  - TUH DWI
    - b=0, … (x7)
- Number of sample data
  - 1,000,000 = 10^6
- Training noise ratio (TNR=$\frac{\sigma}{S_0}$)
  - 0.0, 0.001, 0.01, 0.1
- Validation data noise ratio
  - Same as TNR

Hyperparameters

- Mid Layer #
  - 3, 4, 5
- Mid Layer Units #
  - 64, 128, 256
- Dropout ratio
  - 0.0, 0.01, 0.1
- Epoch #
  - 50, 100, 150
- Batch #
  - 1000, 10000, 100000

The brute force method was used for searching the optimal combination of the hyperparameters.

#3 Example of Training and Tuning: tuned with TNR=0.1 diffusion coefficient D_{x1k}

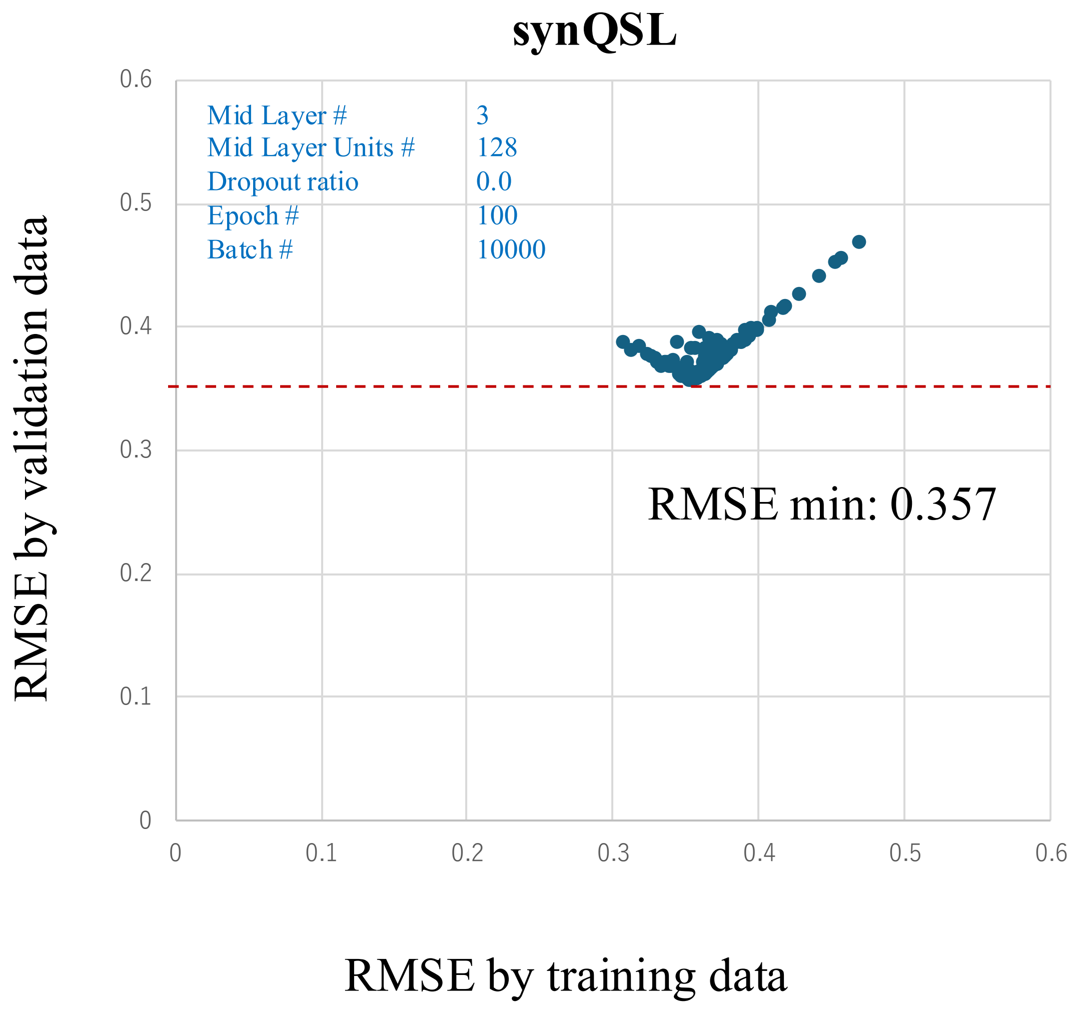

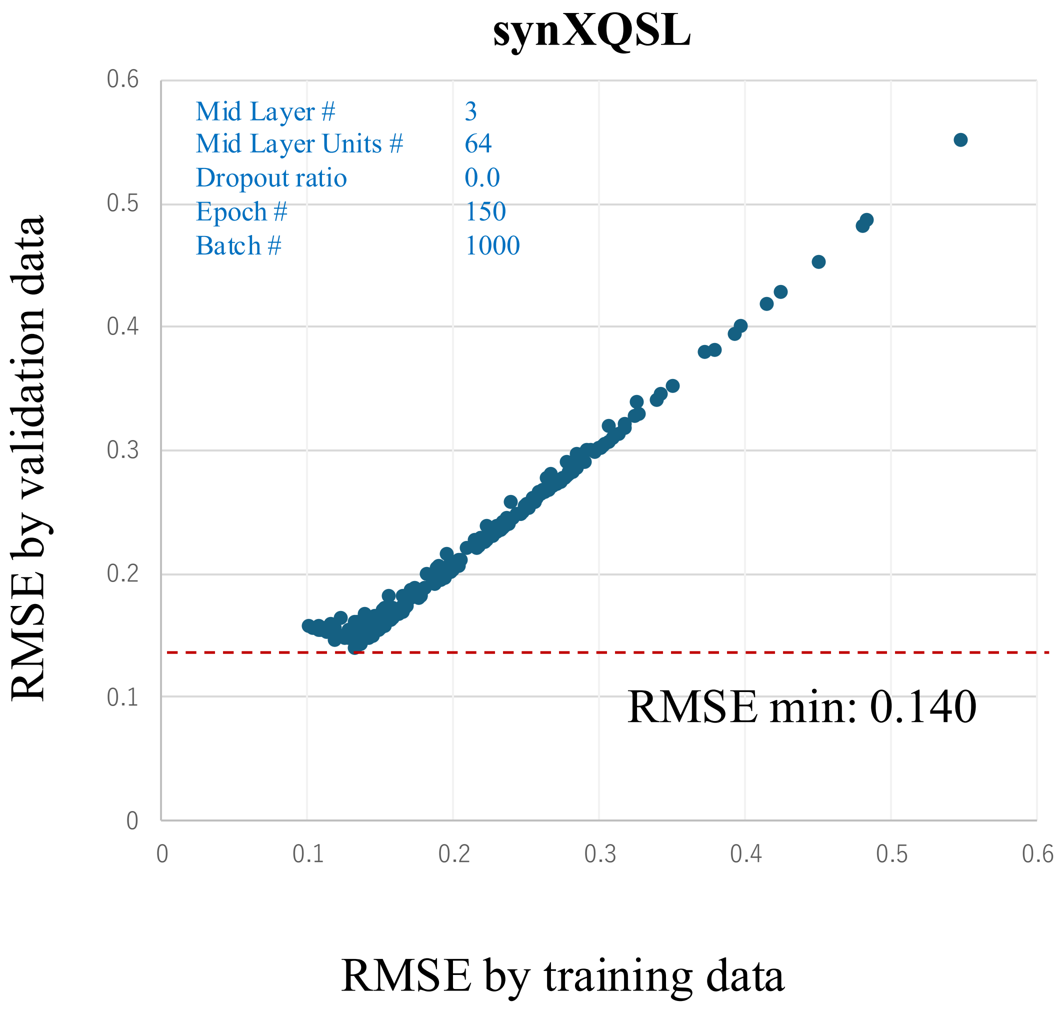

#4 Example of Training and Tuning: tuned with TNR=0.1 diffusional kurtosis *K*

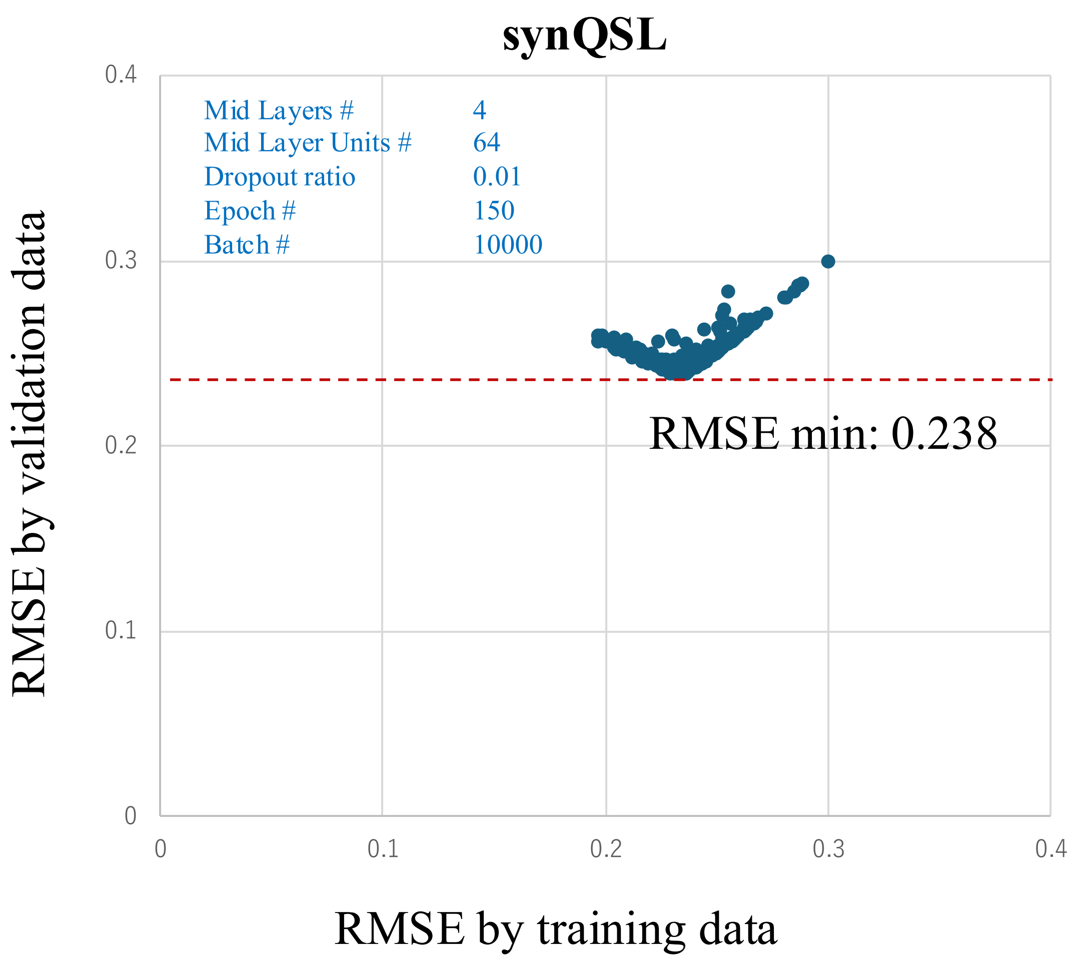

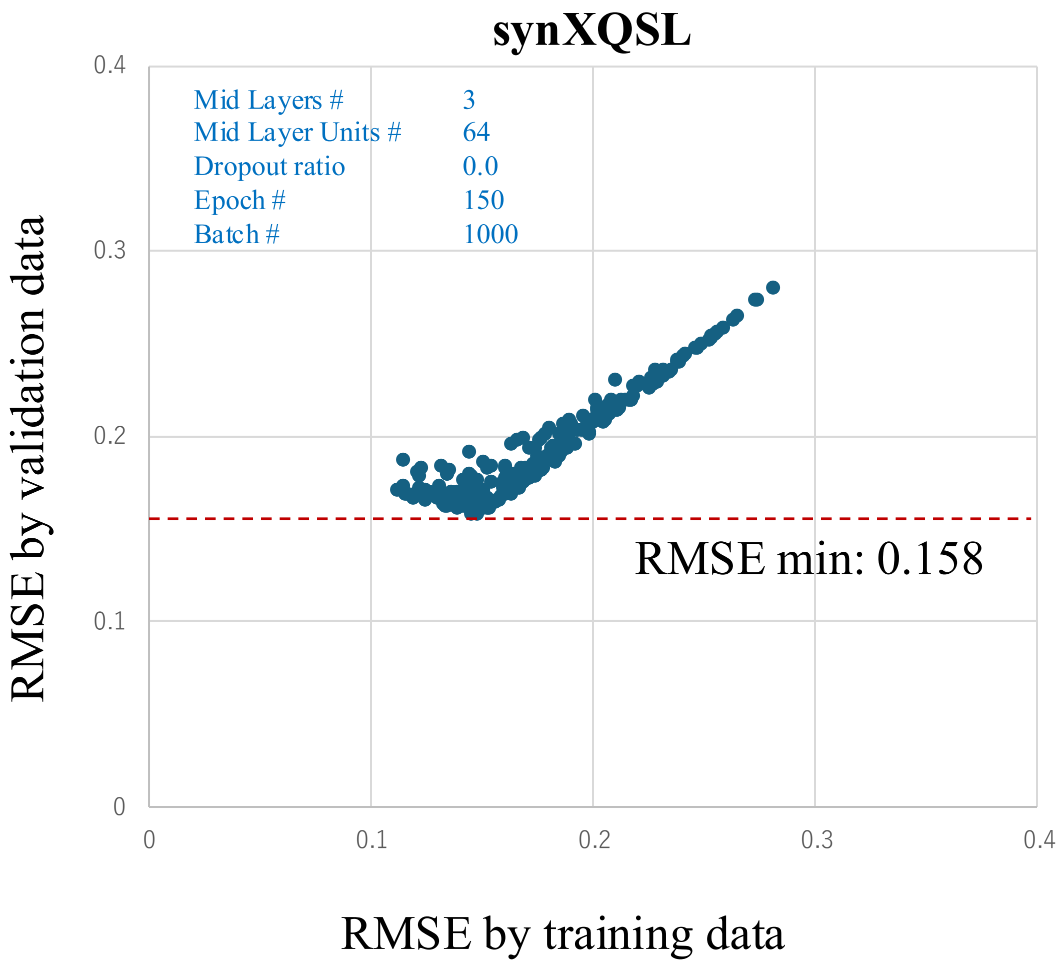

#5 Example of Training and Tuning: tuned with TNR=0.01 diffusion coefficient D_{x1k}

synQSL

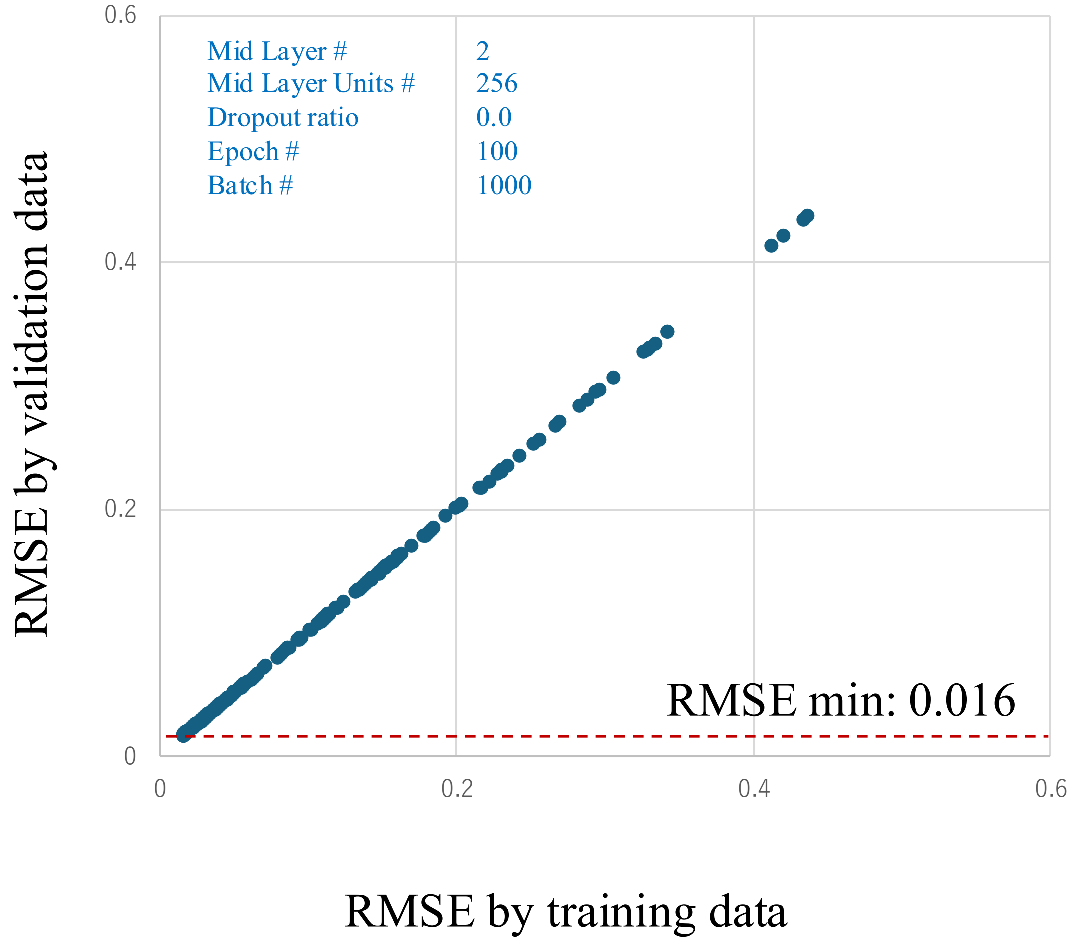

synXQSL

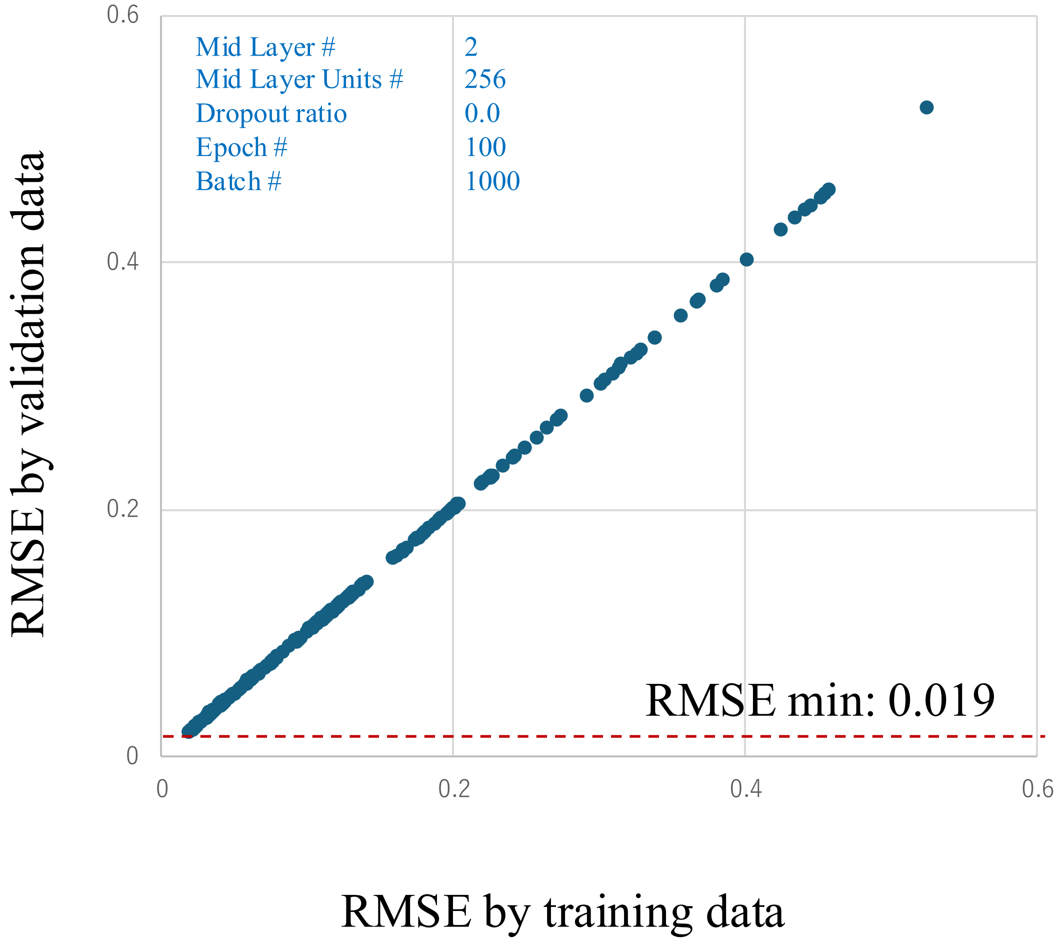

#6 Example of Training and Tuning: tuned with TNR=0.01 diffusional kurtosis *K*

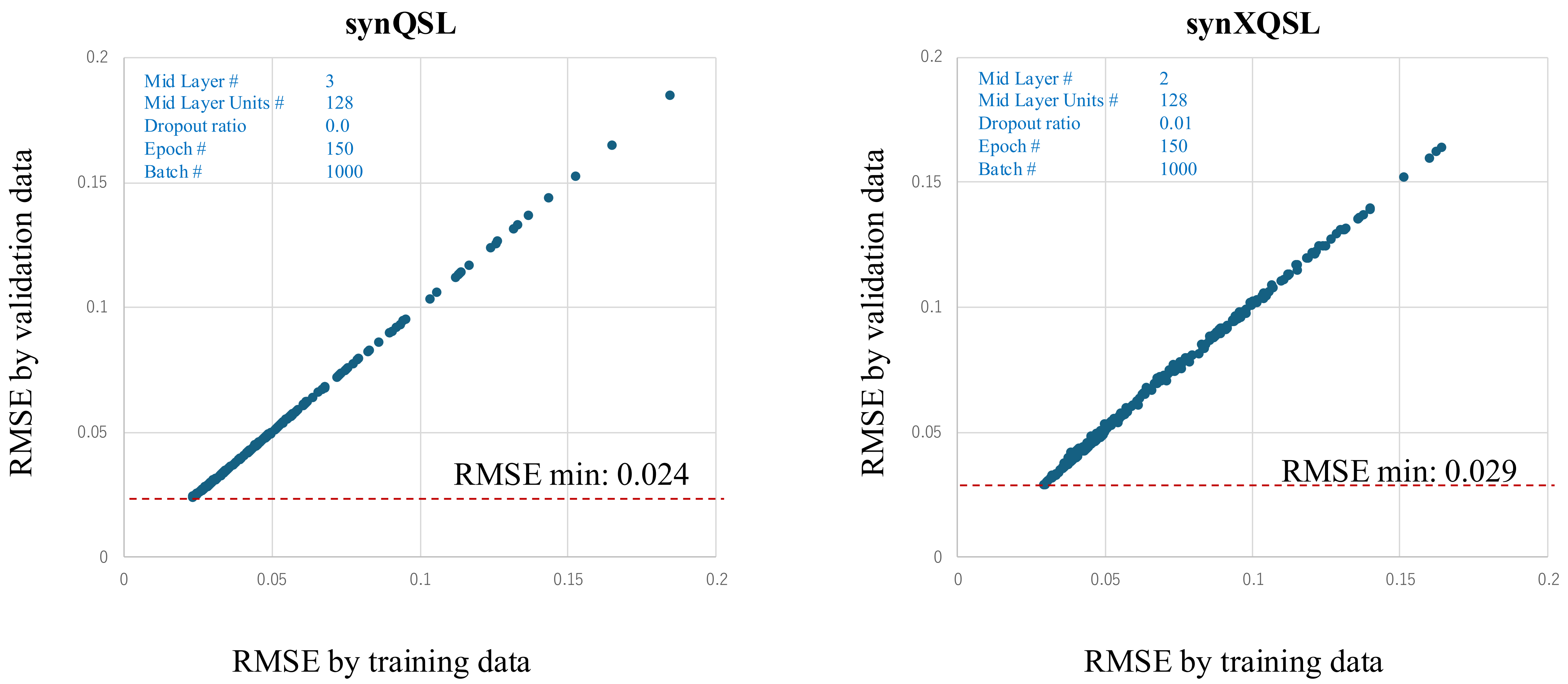

#7 Clinical dMRI Data Example

- Example of clinical dMRI dataset used in this study

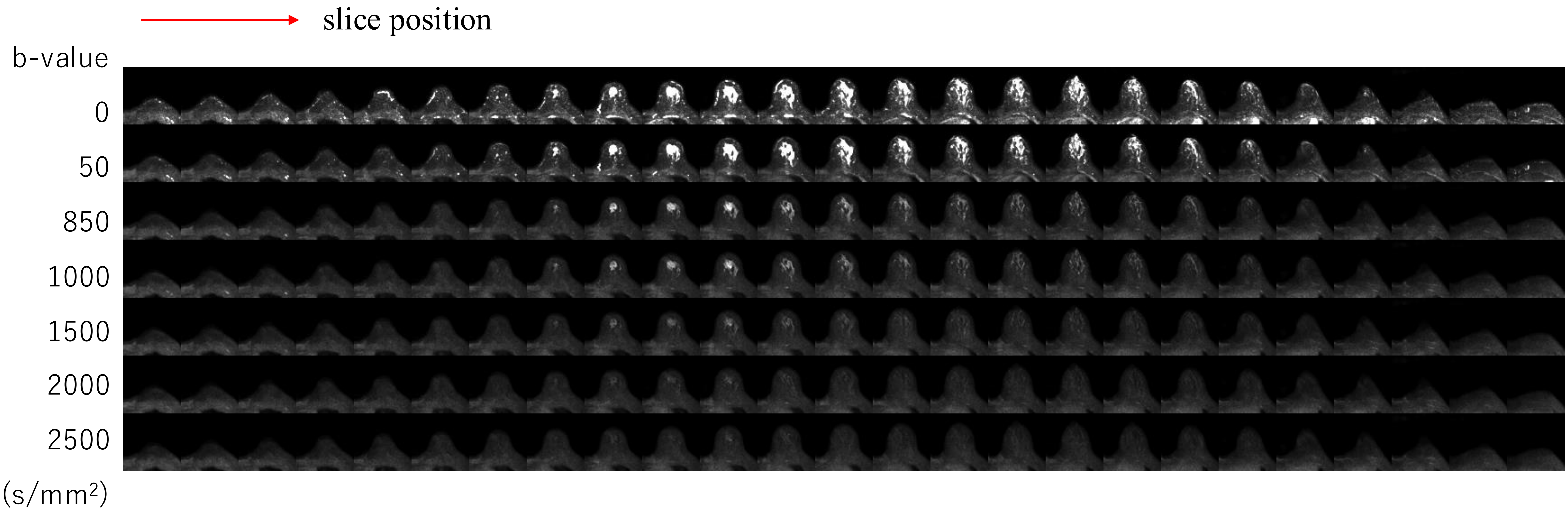

#8 Masks for the quantitative analyses

- For measurement of
  - RMSE, SNR, CNR, and SSIM in digital phantom, and
  - CNR in clinical images

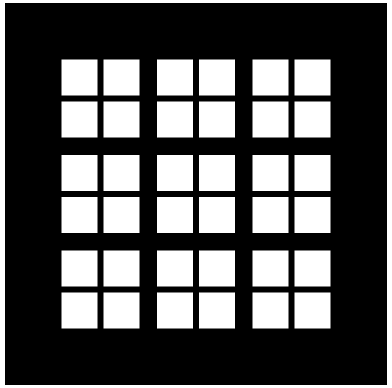

$K \neq 0$
- region for measuring RMSE and SNR
- foreground for CNR

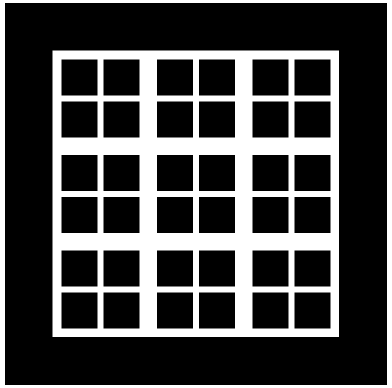

$K = 0$ and $D \neq 0$
- background for CNR

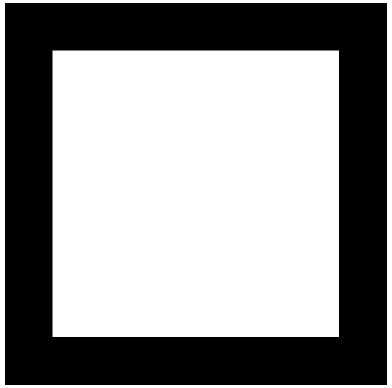

$S_0 \neq 0$
- region for measuring SSIM

digital phantom masks

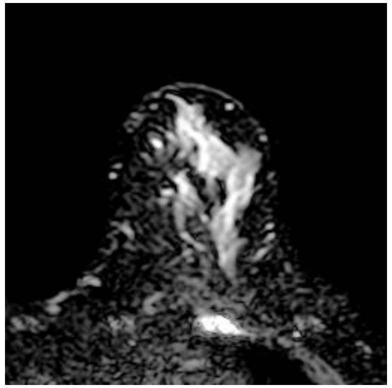

original image
(b=0 DWI)

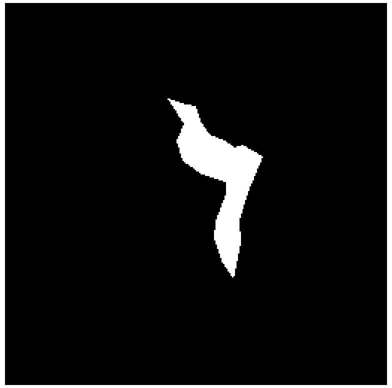

mammary tissues
foreground for CNR
(manually set)

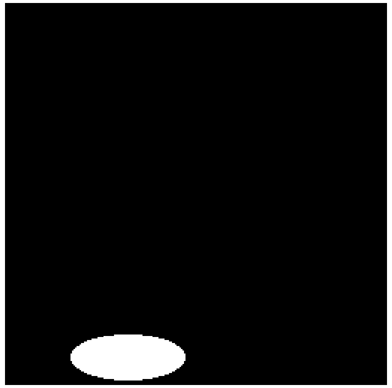

fat tissues
background for CNR
(manually set)

clinical image mask examples

#9 Parameter estimation RMS errors by LSF for synthetic test data

- Note that K estimation for $NR = 0.1$ yielded extremely large RMSE.

Test data noise ratio	RMSE for D $\times 10^3$ (mm^2/s)	RMSE for K
0.000	2.9×10^{-4}	4.1×10^{-7}
0.001	2.4×10^{-1}	3.6×10^{-2}
0.010	3.5×10^{-1}	5.1×10^{-1}
0.100	8.9×10^{-1}	1.8×10^{6}

#10 Results by LSF

- Lower contrast, loss of fine structure and more errors were observed in comparison with the results by synQSL and synXQSL

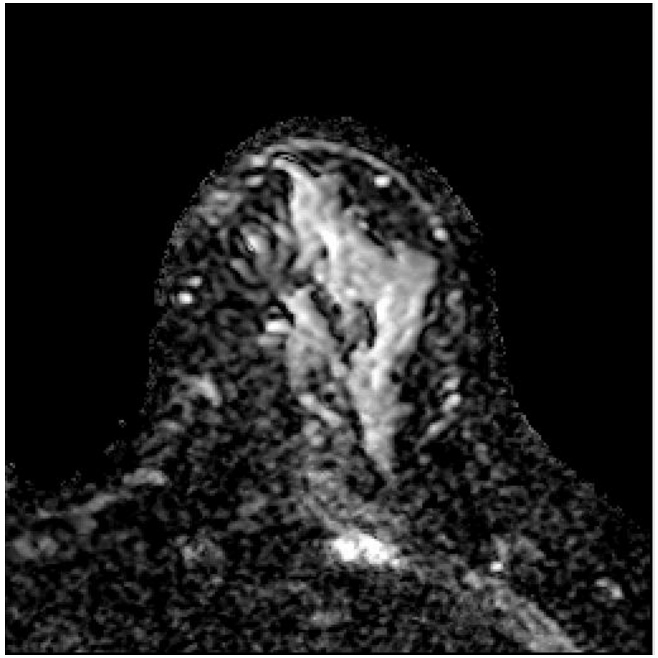

D map

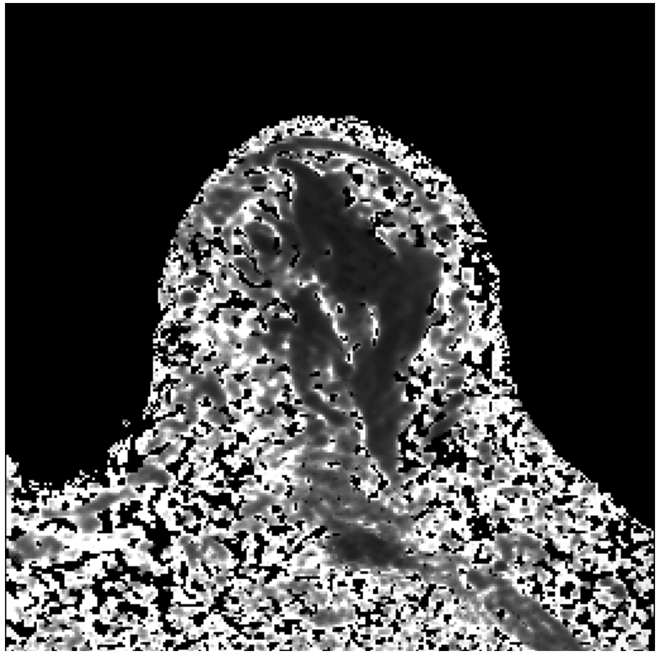

K map

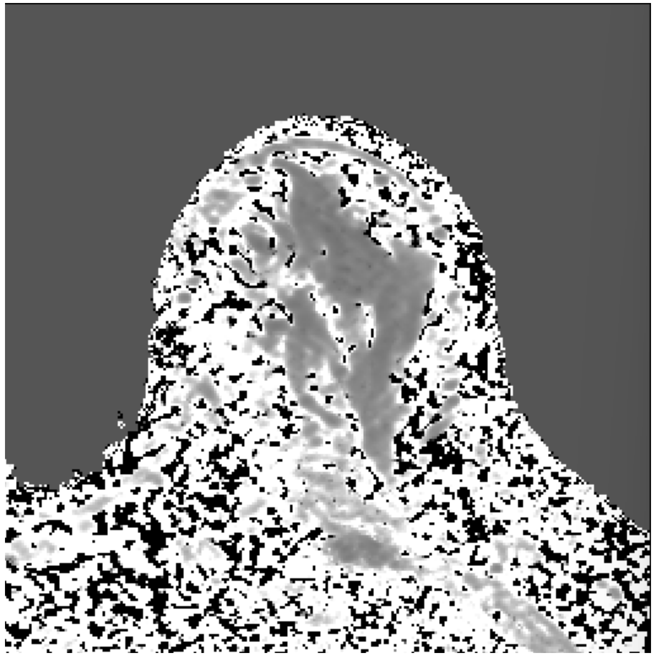

K map
(brightness/contrast adjusted for observation of low *K* area)
